# Security under Uncertainty: Adaptive Attackers Are More Challenging to Human Defenders than Random Attackers

**DOI:** 10.3389/fpsyg.2017.00982

**Published:** 2017-06-22

**Authors:** Frédéric Moisan, Cleotilde Gonzalez

**Affiliations:** ^1^Economics Department, University of CambridgeCambridge, United Kingdom; ^2^Dynamic Decision Making Laboratory, Department of Social and Decision Sciences, Carnegie Mellon UniversityPittsburgh, PA, United States

**Keywords:** defense strategies, security games, game theory, human behavior, learning

## Abstract

Game Theory is a common approach used to understand attacker and defender motives, strategies, and allocation of limited security resources. For example, many defense algorithms are based on game-theoretic solutions that conclude that randomization of defense actions assures unpredictability, creating difficulties for a human attacker. However, many game-theoretic solutions often rely on idealized assumptions of decision making that underplay the role of human cognition and information uncertainty. The consequence is that we know little about how effective these algorithms are against human players. Using a simplified security game, we study the type of attack strategy and the uncertainty about an attacker's strategy in a laboratory experiment where participants play the role of defenders against a simulated attacker. Our goal is to compare a human defender's behavior in three levels of uncertainty (Information Level: Certain, Risky, Uncertain) and three types of attacker's strategy (Attacker's strategy: Minimax, Random, Adaptive) in a between-subjects experimental design. Best defense performance is achieved when defenders play against a minimax and a random attack strategy compared to an adaptive strategy. Furthermore, when payoffs are certain, defenders are as efficient against random attack strategy as they are against an adaptive strategy, but when payoffs are uncertain, defenders have most difficulties defending against an adaptive attacker compared to a random attacker. We conclude that given conditions of uncertainty in many security problems, defense algorithms would be more efficient if they are adaptive to the attacker actions, taking advantage of the attacker's human inefficiencies.

## Introduction

Security problems involve offensive and defensive actions across nations, institutions, and individuals. Attackers aim at stealing and getting access to assets, information, and goods while defenders allocate their limited security resources to prevent attackers from stealing their goods. In home security for example, a home owner may assign alarm systems to strategic locations of the house. Yet, attackers foreseeing the way home owners behave, may be able to find simple and unexpected ways to break into the house (i.e., thorough the front door). Defending against intelligent unauthorized intrusions in the cyber world can be even more challenging, given the hyper-dimensionality of the environment, the type of digital weapons used, the speed of operations and large number of nodes to protect against a relative high number of potential attackers (Gonzalez et al., 2015). In this research we address a basic question of how do human defenders behave under several levels of uncertainty and various types of attack strategies.

Game theory is a common formalized way to inspire the development of defense algorithms in several security problems (e.g., Roy et al., 2010; Tambe, 2011; Shieh et al., 2012). For example, Stackelberg games (i.e., strategic sequential games in which one's own strategy is reactive to one's rival's actions), are common in the design of algorithms that help allocating limited security resources, and results have been successfully applied to multiple naturalistic settings (e.g., Jain et al., 2010; Fang et al., 2013). Although these strategies have been mostly demonstrated in the physical world, a parallel situation occurs in the cyber world, where there is a need of protecting from electronic criminal activities. Researchers have also turned to using game theory to understand security and defense strategies in the cyber world (Alpcan and Başar, 2010; Dutt et al., 2013; Gonzalez, 2013). Attacks in the cyber world (i.e., cyberattacks) use digital weapons that are often imperceptible to the human senses; they are not limited by geography and political boundaries; they require of highly sophisticated technical knowledge, and they may be highly dynamic and distributed. Thus, a defender in the cyber world may need strategies that are dynamic and adaptive to sophisticated attackers, in contrast to currently common static and non-adaptive defense algorithms (Bier and Azaiez, 2008; Abbasi et al., 2015a,b).

To build effective dynamic and adaptive defense algorithms we need to address at least two strong assumptions in the science of security games (Nguyen et al., 2016) and behavioral game theory more generally (Gonzalez et al., 2015): information certainty and human rationality. Current defense algorithms inspired by game theory assume that a defender has perfect information about the payoff matrix and the attacker's behaviors. They also often assume that players are perfectly rational and are able to account and process all information accurately. In a review of learning models from behavioral game theory, Camerer (2003) concludes that most models rely on a full information assumption, and they would not be able to predict behavior in conditions where this information is not available. Similarly, most of these models make cognitively implausible assumptions, such as players being able to remember large amounts of information (Gonzalez et al., 2015).

This paper contributes to understanding how to possibly address these two challenges by learning from a laboratory experiment how human defenders deal with information uncertainty in games in which the attacker algorithm is random, conservative (i.e., minimizes losses) or adaptive (i.e., adapts to the defender's behavior), using an adversarial security game with payoff asymmetry that mimics real life interactions between a defender and an attacker. The conclusions that we draw from our study are general in nature and have applications to several security problems. We discuss the implications our results have for the design of defense algorithms against adaptive attackers under conditions of uncertainty.

### An asymmetric cybersecurity game, attack algorithms, and information uncertainty

**Figure 1:**
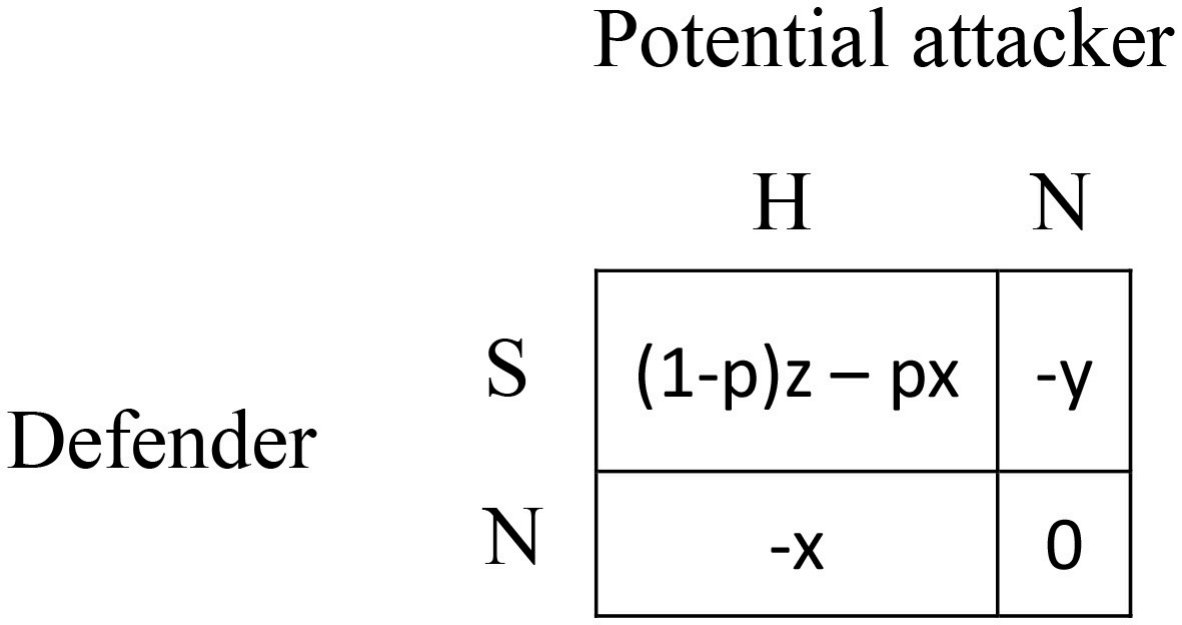
Defender's payoffs in generic asymmetric security game (0 < y < x, 0 < z, 0 < p < 1).

Figure 1 illustrates a generic non-cooperative game with two-players, each of them being able to take two possible actions. In the context of cybersecurity, attacker, and defender interact in non-cooperative ways: The players have conflicting interests (one's gains correspond to the other's losses) characterized by a zero-sum property. In other words, there is no value in cooperating in such interactions because no player can win without making the other one lose. Furthermore, the game is fully strategic since any player's best move strictly depends on the other player's move.

Consider a simple strategic game where a potential attacker may either hack the defender's network (H) or do nothing (N) and a defender that may either surveil the network (S) or do nothing (N). In such a scenario, there exists some probability p (0 < p < 1) that the hack is successful despite surveillance. This means that there is a probability 1 − p that the hack has been stopped. The Appendix provides a game-theoretic analysis of this scenario and discusses the various assumptions of the outcomes from the strategic actions (x, y, and z), as well as the assumptions regarding the probability of the hacker's success (p).

Experimentally controlling for different attack strategies while investigating human defenders, is a common research approach (Lieberman, 1962; Messick, 1967; Fox, 1972; Coricelli, 2004; Shachat and Swarthout, 2004, 2012; Dürsch et al., 2006; Spiliopoulos, 2008). In competitive scenarios, there often exist well-known simple strategies that vary in the level of rationality and level of adaptation to the defender's behavior. A *Random* strategy (Fox, 1972) is a fixed and static strategy consisting of choosing every option with an equal probability. Such rule is independent of the payoff matrix and the defender's behavior. A *Minimax* strategy implies the minimization of the possible loss of a worst case scenario (Lieberman, 1962; Messick, 1967; Fox, 1972; Shachat and Swarthout, 2004). When defined as a mixed strategy in a two-player zero-sum game, this principle guarantees the same expected payoff independently of the defender's choice (see Appendix). An *Adaptive* strategy may imply that an attacker accounts for the history of the defender's actions, estimates the defender's most likely move in the current round and choose a best response accordingly (Brown, 1951; Robinson, 1951; Messick, 1967; Dürsch et al., 2006). Unlike the other strategies, an Adaptive strategy takes into account the other player's past behavior to decide on the own agent's current behavior. The specific adaptive strategy that we consider in this study corresponds to the well-known fictitious play (Brown, 1951; Robinson, 1951).

Uncertainty regarding the attacker's actions and outcomes in repeated strategic interactions is of particular interest in cybersecurity and the lack of explicitly described information naturally leads people to rely more on their own past experience. Risky behaviors may be contrastingly different in situations where people rely on descriptions or experience to make decisions (Hertwig et al., 2004). This body of research suggests fundamental psychological differences in how humans make decisions under uncertainty (Hau et al., 2008; Hertwig and Erev, 2009). In behavioral game theory, uncertainty effects are also of great interest (Martin et al., 2014; Gonzalez et al., 2015). For example, Bereby-Meyer and Roth (2006) have studied players' abilities to learn cooperation in a repeated PD game where the payoff descriptions were more or less noisy: in each outcome, the payoffs were presented either deterministically or probabilistically. The speed of individual learning was substantially diminished when the payoffs were noisy (non-deterministic), even though players could monitor one another's past actions perfectly. More generally, these studies show that even small changes in payoff environment can have a large effect on collective behavior. Our main general hypothesis is that introducing uncertainty in the attacker's payoffs (through the probability p) can affect the human defender's behavior in different ways depending on the attacker's strategy: uncertainty may lead defenders to follow noisier and more unpredictable behavior unless the attacker's strategy is sufficiently naive and exploitable.

## Methods

We examine the combined effects of (1) the attacker's preprogrammed strategy and (2) the level of uncertainty in the outcome description, on a defender's behavior in the asymmetric security game (Figure 1), using a 3 (Information Level: Certain, Risky, Uncertain) × 3 (Attacker's strategy: Minimax, Random, Adaptive) between-subjects experimental design.

**Figure 2:**
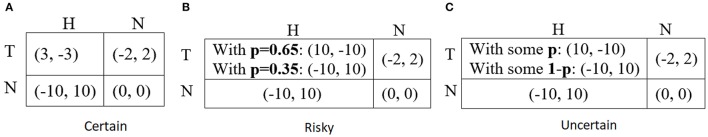
Asymmetric security game in various conditions with x = z = 10, y = 2, and probability *p* = 0.35. **(A)** Shows the certain condition in which participants have complete information about all possible outcomes. **(B)** Shows the risky condition in which participants receive explicit probabilities of possible outcomes. **(C)** Shows the uncertain condition in which participants are not informed about the values of the probability p.

Under Certain information, participants have complete information about all possible outcomes; they receive the game as in Figure 2A, corresponding to a certainty case of the game presented in Figure 1. For example, when the defender chose S and the potential attacker chose N, the outcome (S,N) indicates a payoff of −2 points to the defender and 2 points to the attacker.

Under Risky information (Figure 2B), participants only have partial information about possible payoffs they can obtain as the defender chooses S and the attacker selects H. Under Uncertain information (Figure 2C), participants are never informed about the actual value of probability p that determines the (S, H) consequence. In this case, participants only know that one player will win while the other will lose depending on the unknown probability p. Importantly, the games in all information conditions are theoretically equivalent in the sense that the same cells of the game carry the same expected payoffs: the value of p in Figures 2B,C is 0.35; the deterministic payoffs in the case of the (S,H) outcome from the Certain conditions correspond to the expected payoffs in the same outcome (where *p* = 0.35) in the two other conditions (Risky and Uncertain descriptions).

The attacker's strategy is manipulated into three algorithms: Minimax, Random, and Adaptive. In Minimax, the attacker always follows the Minimax principle by selecting H with $\frac{2}{15}$ probability (and therefore N with $\frac{13}{15}$ probability) in every round (see Appendix). These values derive from the payoffs in the baseline setting of Figure 2A. In this strategic game, the defender's Minimax strategy is to select S with a $\frac{2}{3}$ probability and N with a $\frac{1}{3}$ probability (in each round, the defender's corresponding expected payoff is $-\frac{4}{3}$ and potential attacker's corresponding expected payoff is $\frac{4}{3}$). Note that this particular game allows for a clear distinction between both players' optimal and the Random strategy, which consists of a purely random choice where the attacker selects H or N with equal probability (0.5) at every round. The Adaptive algorithm is as follows: in the very first round of the game, the attacker selects H or N with equal probability (as with the Random strategy), and in all subsequent rounds, its choice is based on the defender's history of past moves (see Appendix).

In all conditions, participants are asked to repeatedly play against the same attacker strategy for 100 rounds (the participants are not informed about the exact number of rounds). At every round *r* > 1, participants receive feedback indicating the actual outcome in the previous round.

### Participants

Nine Hundred and Twenty Seven American individuals (61% male; M_age_ = 30.8, SD_age_ = 9.3) were recruited through Amazon Mechanical Turk. Participants were randomly assigned to one of the 9 different conditions previously described: N(Minimax-Certain) = 107, N(Minimax-Risky) = 99, N(Minimax-Uncertain) = 101, N(Random-Certain) = 103, N(Random-Risky) = 102, N(Random-Uncertain) = 105, N(Adaptive-Certain) = 110, N(Adaptive-Risky) = 100, and N(Adaptive-Uncertain) = 100. Upon completion of the experiment, each person was paid based on their performance in the task. The average time spent to complete the task was 5 min 09 s, and the average amount of total payment was $0.88, including a fixed participation fee of $0.3. This research complied with the American Psychological Association Code of Ethics and the tenets of the Declaration of Helsinki and it was approved by the Institutional Review Board at Carnegie Mellon University. Informed consent was obtained from each participant.

### Procedure

Starting with an initial capital of 200 points, participants were instructed to repeatedly play a game with an unknown preprogrammed attacker strategy, where each round would determine either a gain of extra credits or a loss of points from their capital. They were informed that the immediate outcome of each round depended on their own choice between two options (A and B), as well as on the attacker's decision. Note that the labeling of the players and their actions were different from those shown in Figure 2, in order to avoid uncovering any focal point that may bias people's behavior (“opponent” instead of “attacker,” option “A” instead of “S” or “H,” and option “B” instead of “N”). Participants were told that their income by the end of the experiment (in US dollars) would be calculated based on the value of their remaining total capital (if any) with a conversion rate of US$0.01 for every point.

Participants were not provided with any information regarding the attacker's strategy, except that the attacker was motivated to steal as many points as possible from their capital. They were provided with the payoff matrix corresponding to the experimental condition (as in Figure 2). The display of the payoff matrix was determined randomly from four combinations of actions [i.e., (A,A), (A,B), (B,A), or (B,B)] to control for possible effects of the game's display and the buttons' labels. In every round, feedback was provided about what choice each player made in the previous round together with the resulting payoffs.

## Results

**Figure 3:**
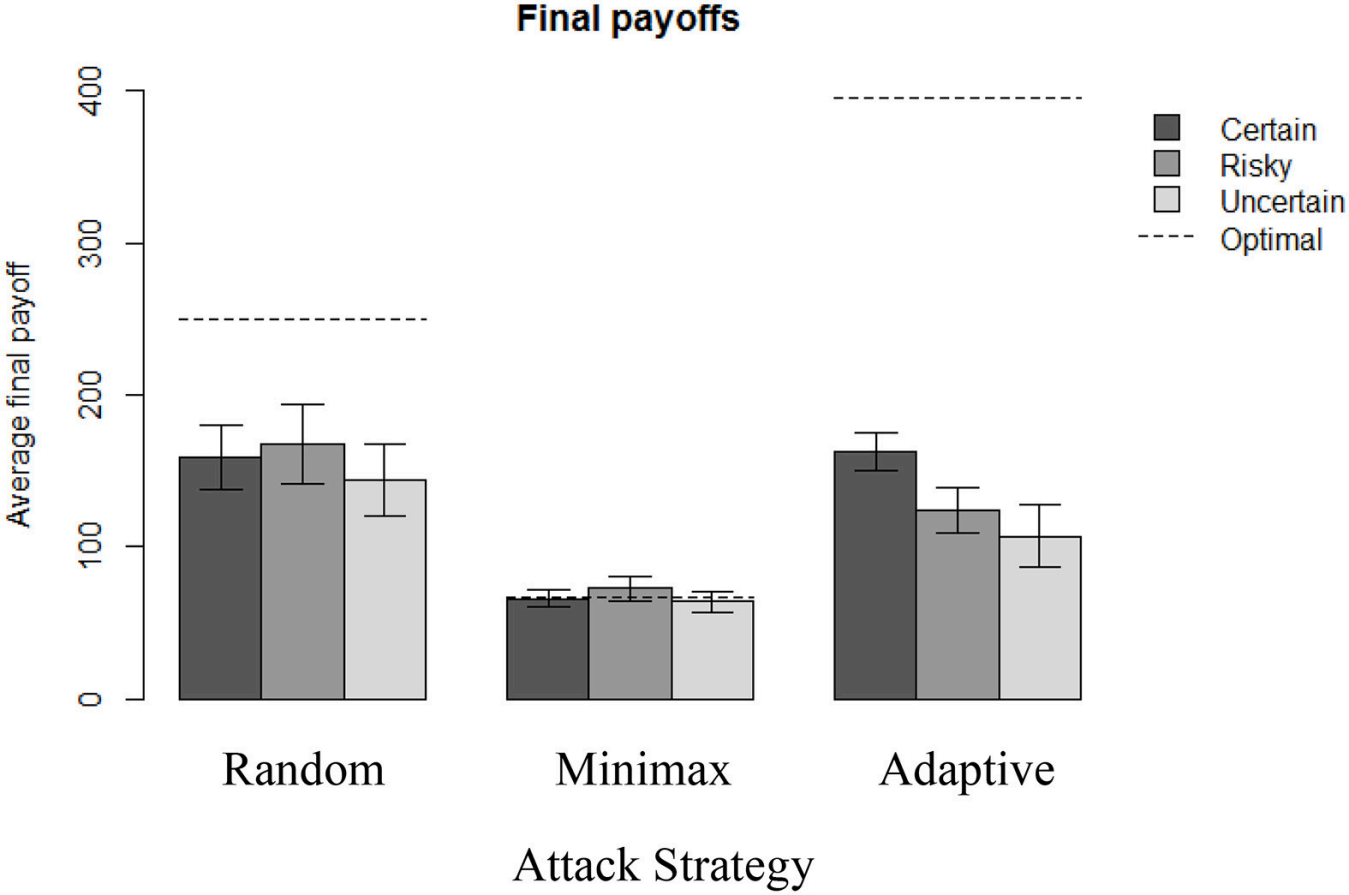
Final payoffs in each condition across 100 rounds. Initial endowment = 200 pts. The dotted lines represent the corresponding final payoff when the optimal solution is applied consistently.

The maximum expected final payoff for the defender was obtained when playing optimally against the Adaptive strategy (395.5 pts, see Appendix), and playing against Random (250 pts) or Minimax (66.7 pts) strategies was less beneficial. Figure 3 provides a summary of the corresponding final payoffs that participants obtained in each condition. Note that although participants did not lose money in this experiment (any final negative final payoff was simply reduced to a zero gain), the values used in Figure 3 are based on raw data that potentially include negative final payoffs.

All payoffs can be compared to a reference payoff of 66.7 pts, which can be guaranteed to the defender against the Minimax strategy in every round, because the game is zero-sum. We observe in Figure 3 that this reference payoff is reached under the Minimax strategy in all uncertainty conditions. Average final payoffs from all conditions are at least as good as this reference payoff. Also, defenders' behavior appear better when the attacker's strategy is Adaptive or Random, compared to Minimax. However, the defenders' behavior is suboptimal compared to the maximum expected payoff they could have obtained against a Random and Adaptive strategy over 100 rounds.

Looking at the interaction effect between the attacker's strategy type and the level of uncertainty on the participant's final payoff, Table 1 provides evidence that those factors significantly influence people's payoffs in the game (after playing 100 rounds).

**Table 1:** Factorial analysis of variance for final payoffs.

**Source**	**ANOVA tests**		
	**D*f***	***F***	**Eta-Squared**
(A) Attacker's strategy	2	82.39^**^	0.15
(B) Outcome uncertainty	2	6.11^*^	0.01
A × B (interaction)	4	3.44^*^	0.01
Error (within groups)	918		

** *p < 0.001*;

* *p < 0.01*.

Figure 3 and Table 1 reveal an effect of the attacker's strategy: the best final payoffs are obtained when playing against Random (*N* = 310, *M* = 156 pts, *SD* = 125), which are significantly better than when playing against Adaptive [*N* = 310, *M* = 132 pts, *SD* = 87; two sample *t*-test: *t*_(552)_ = 2.8, *p* < 0.01, *d* = 0.22]. Playing against Adaptive is, however, still more significantly beneficial than playing against Minimax [*N* = 307, *M* = 67 pts, *SD* = 35; two sample *t*-test: *t*_(408)_ = 12, *p* < 0.001, *d* = 0.98]. However, outcome uncertainty is also significant: playing in the Certain condition (*N* = 320, *M* = 129 pts, *SD* = 89) is significantly more profitable than playing in the Uncertain condition [*N* = 306, *M* = 105 pts, *SD* = 101; two sample *t*-test: *t*_(606)_ = 3.1, *p* < 0.01, *d* = 0.25]. The interaction effects presented in Figure 3 suggest that uncertainty only has a significant effect on the overall payoff when playing against Adaptive strategy. Increasing uncertainty significantly decreases efficiency: final payoffs in the Adaptive-Certain condition (*N* = 110, *M* = 163 pts, *SD* = 68) are significantly better than Adaptive-Risky [*N* = 100, *M* = 124 pts, *SD* = 78; two sample *t*-test: *t*_(198)_ = 3.8, *p* < 0.001, *d* = 0.53] and Adaptive-Uncertain [*N* = 100, *M* = 107 pts, *SD* = 104; two sample *t*-test: *t*_(169)_ = 4.5, *p* < 0.001, *d* = 0.64]. On the other hand, increasing uncertainty has no significant effect when playing against Random and Optimal.

Moreover, when the payoffs are all known and deterministic, people are as efficient against Random as against Adaptive (*M* = 159 pts in Random-Certain condition, and *M* = 163 pts in Adaptive-Certain condition). One could even argue that people play better against Adaptive because of the slightly higher final payoff on average and the smaller standard deviation (*SD* = 113 in Random-Certain condition, and *SD* = 68 in Adaptive-Certain condition). This observation is particularly surprising because of the very different complexity in computing the best response in both of these conditions: learning to play optimally against Random (by always playing S) is easier as it is shown when risky or uncertain information is introduced.

### Choice behavior

**Figure 4:**
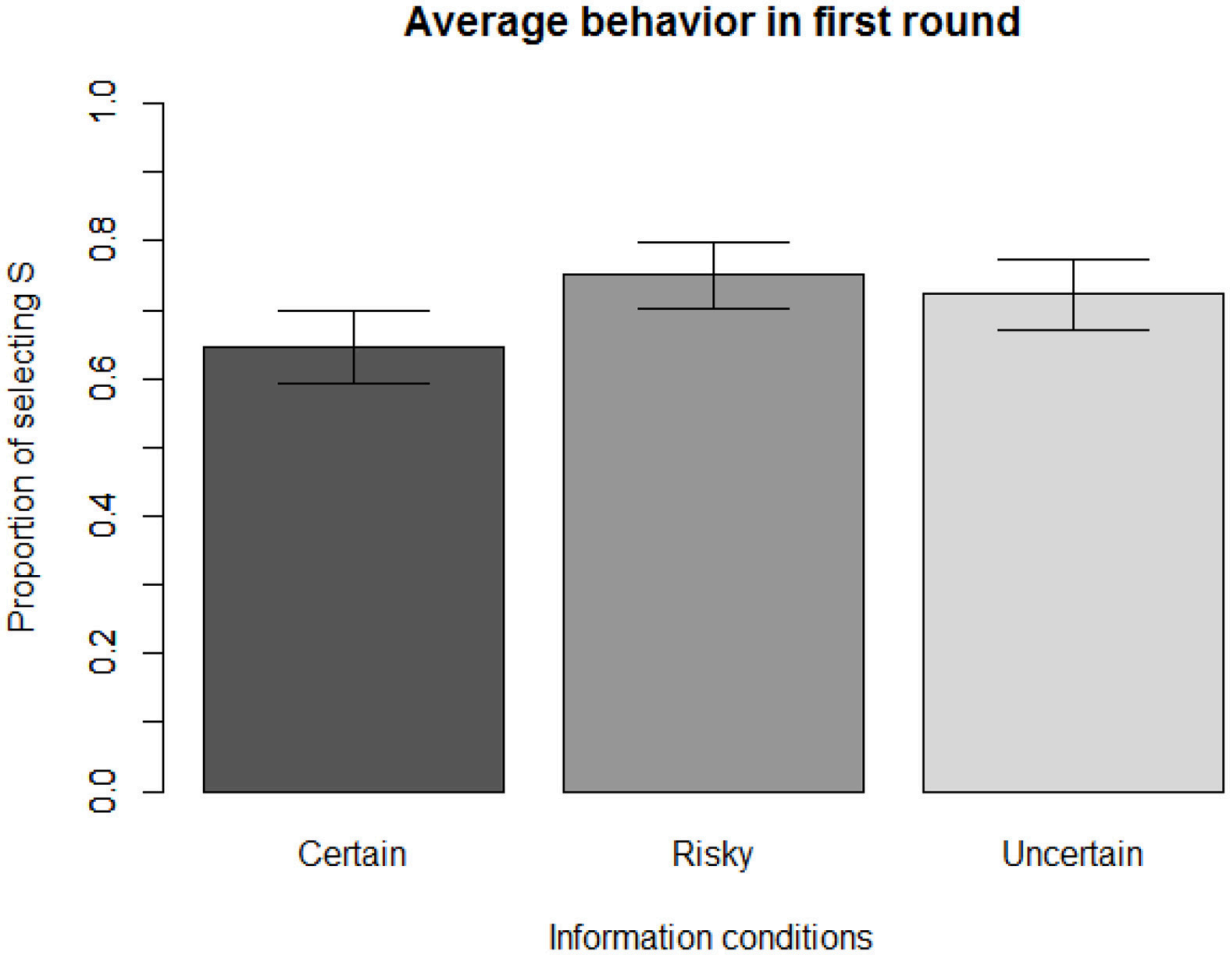
Average initial behavior across information conditions.

Figure 4 shows that playing the game in the Certain condition leads to an average behavior that closely approaches the prediction of the Optimal principle (selecting S with a $\frac{2}{3}$ probability, as shown earlier). The rate of selecting S in the Certain condition is 65% (*N* = 320), which is not significantly different from the theoretical Minimax solution of 66.66% [*t*_(319)_ = −0.74, *p* = 0.46, *d* = 0.08].

However, participants' behavior differed significantly in the first round [*F*_(2, 616)_ = 4.22, *p* = 0.015). Introducing some uncertainty in the (S,H) outcome leads to a significant increase in the rate of selecting S: 75% of participants (*N* = 301) chose S during the first round in the Risky condition, which is significantly different from the Certain condition at the 1% level [*t*_(618)_ = −2.84, *p* = 0.005, *d* = 0.23]. Similarly, 72% of participants (*N* = 306) chose S during the first round in the Uncertain condition, which is significantly different from the Certain condition at the 5% level [*t*_(624)_ = −2.03, *p* = 0.04, *d* = 0.16].

Table 2 shows the effect of the attacker's strategy. Uncertainty level has no significant effect. Results indicate a significant effect of rounds on average behavior, but the interaction between uncertainty and attacker's strategy is not significant.

**Table 2:** Factorial analysis of variance for average choice over blocks of 10 rounds.

**Source**	**ANOVA tests**			
	**D*f***	**D*f*_error_**	***F***	**Eta-Squared**
(A) Attacker's strategy	2	918	245.24^**^	0.21
(B) Outcome uncertainty	2	918	0.89	<0.01
(C) Blocks of 10 rounds	7.43	6,820.28	9.45^**^^†^	<0.01
A × B (interaction)	4	918	0.93	<0.01
A × C (interaction)	14.86	6,820.28	14.04^**^^†^	0.01
B × C (interaction)	18	8,262	1.42	<0.01
A × B × C (interaction)	36	8,262	1.00	<0.01

** *p < 0.001*;

† *Greenhouse, Geisser corrected*.

**Figure 5:**
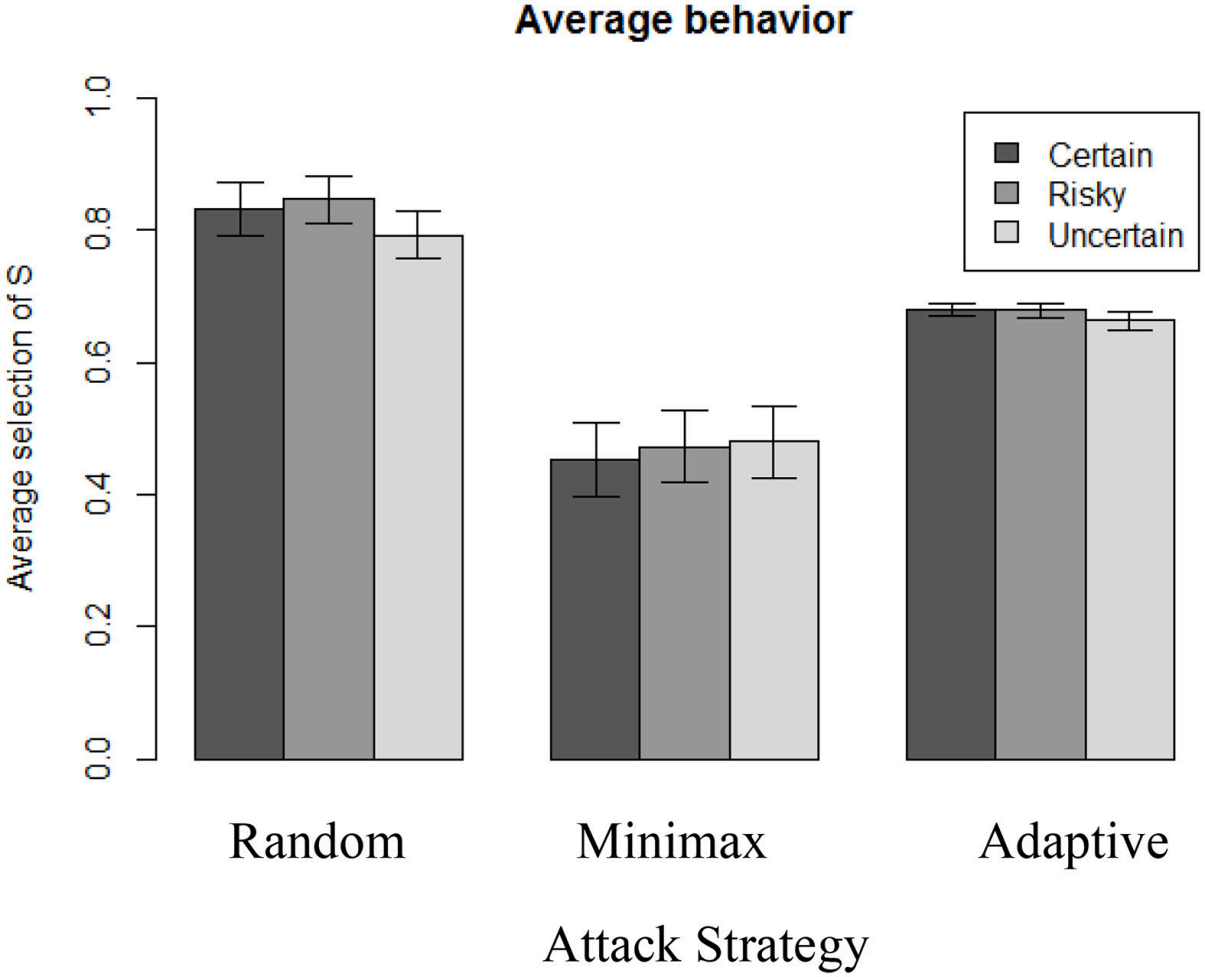
Average selection of S action in each condition.

Figure 5 illustrates the differences in overall average choices depending on the attacker's strategy over the 100 rounds, and also reveals different levels of heterogeneity in individual behavior across conditions. The highest proportion of selecting S is reached when playing against Random (*N* = 310, *M* = 82%, *SD* = 19), which is significantly higher than when playing against the Adaptive [*N* = 310, *M* = 67%, *SD* = 6; *t*_(364)_ = 12.9, *p* < 0.001, *d* = 1.06]. Playing against Adaptive, however, leads to a more frequent selection of S than when playing against the Minimax strategy [*N* = 307, *M* = 47%, *SD* = 28; *t*_(332)_ = 12.6, *p* < 0.001, *d* = 0.99].

**Figure 6:**
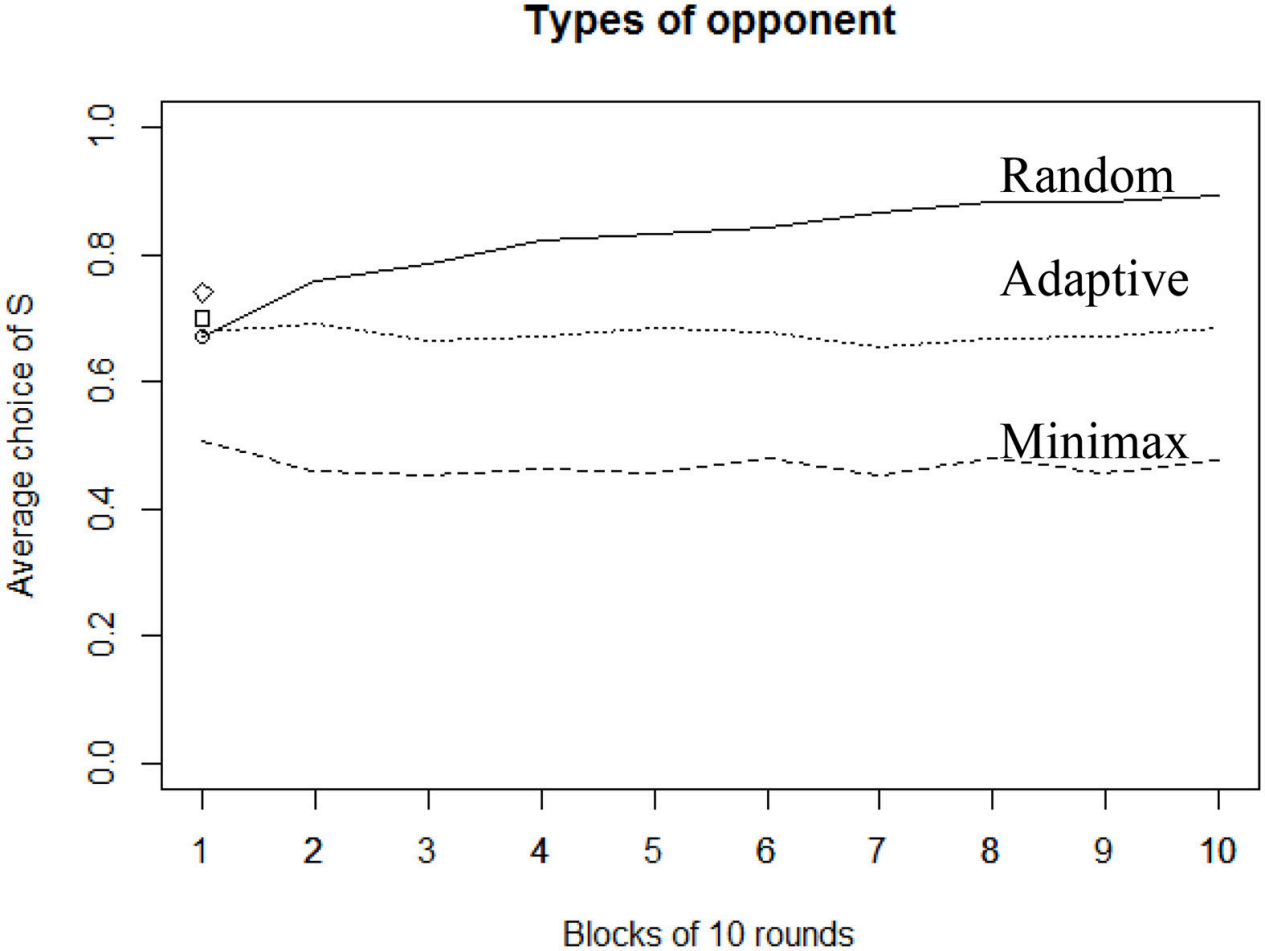
Average proportion of choice (S) over time. Each block is 10 rounds.

Figure 6 depicts the overall effect of the number of rounds over the proportion of S choices. The main observation is that only the Random opponent's strategy leads defenders to adapt and increase their proportion of selecting S over time. In this case, participants learn to play the best response against the Random strategy (which corresponds to always selecting S). Also, average behavior drifts away from the theoretical Minimax solution (playing S with probability 0.67). Instead, when playing against the Minimax opponent strategy, we observe rather stable behavior across time. Average selection of S is above 65% in the first round and drops to 44% within the first 20 rounds before stabilizing for the remaining rounds. As a consequence, during the very first rounds, people's behavior again drifts away from the theoretical Minimax solution, but in a different direction as compared to the previous condition (playing against Random). This observation indicates that people do not learn to minimize the variance of their payoff (which consists in always playing T). Instead, they quickly become more indifferent between their options, regardless of the level of uncertainty.

Finally, when playing against Adaptive strategy, there is no learning effect. The standard deviation of the attacker's average behavior is, however, larger than in the Random condition (SD in Adaptive = 19; SD in Random = 5), suggesting that react to Adaptive strategy, but do not follow an adaptive strategy themselves (if they did, the attacker's average behavior would then converge toward the Nash equilibrium).

### Switching behavior

**Figure 7:**
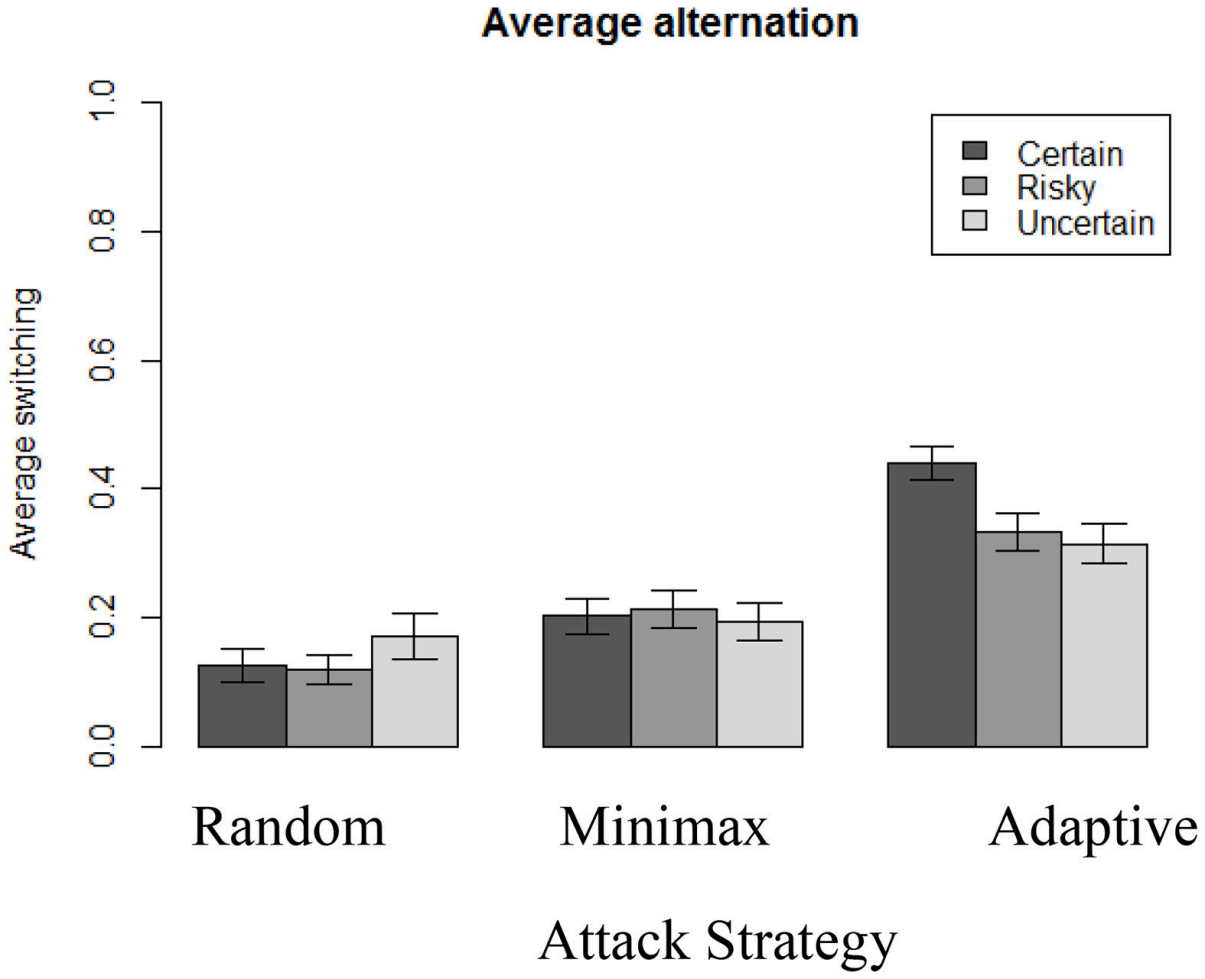
Average switching behavior in each condition.

We analyzed the number of times that participants switch decisions from one option to the other in the course of 100 rounds. This analysis is relevant as it is possible that two individuals who share the same choice proportion of S reach that stage through different exploration strategies. As shown in in Table 3, there is a clear effect of both outcome uncertainty and the attacker's strategy on switching behavior. Furthermore, the interaction effect between the two factors is significant, as illustrated in Figure 7.

**Table 3:** Factorial analysis of variance for switching behavior over blocks of 10 rounds.

**Source**	**ANOVA tests**			
	**D*f***	**D*f*_error_**	***F***	**Eta-Squared**
(A) Attacker's strategy	2	918	191.20^**^	0.17
(B) Outcome uncertainty	2	918	5.06^**^	<0.01
(C) Blocks of 10 rounds	7.40	6,791.28	20.09^**^^†^	0.01
A × B (interaction)	4	918	10.52^**^	0.02
A × C (interaction)	14.80	6,791.28	16.96^**^^†^	0.02
B × C (interaction)	18	8,262	0.76	<0.01
A × B × C (interaction)	29.59	6,791.28	2.01^**^^†^	<0.01

** *p < 0.001*;

† *Greenhouse, Geisser corrected*.

Switching occurs more often when playing against Adaptive (*N* = 310, *M* = 36%, *SD* = 16) than when playing against Minimax [*N* = 307, *M* = 20%, *SD* = 15; *t*_(614)_ = 13.06, *p* < 0.001, *d* = 1.03], which is also significantly more than when playing against Random [*N* = 310, *M* = 14%, *SD* = 15; *t*_(615)_ = 5.38, *p* < 0.001, *d* = 1.42]. We also observe a main effect of uncertainty: switching in the Certain condition (*N* = 320, *M* = 26%, *SD* = 19) is higher than in the Risky condition [*N* = 301, *M* = 22%, *SD* = 17; *t*_(613)_ = 2.64, *p* < 0.01, *d* = 0.22] and the Uncertain condition (*N* = 306, *M* = 22%, *SD* = 17; *t*_(622)_ = 2.64, *p* = 0.02, *d* = 0.22]. These results suggest an interaction effect of the two variables (type of attacker strategy and outcome uncertainty). Indeed, when playing against Random, participants alternate significantly more (at the 5% level) under the Uncertain condition (*N* = 103, *M* = 17%, *SD* = 18) than under the Certain condition [*N* = 105, *M* = 12%, *SD* = 13; *t*_(190.6)_ = 2.08, *p* = 0.039, *d* = 0.32]. On the other hand, when playing against Adaptive, participants alternate significantly less under the Uncertain condition (*N* = 100, *M* = 31%, *SD* = 15) than under the Certain condition [*N* = 110, *M* = 44%, *SD* = 14; *t*_(201.3)_ = 6.12, *p* < 0.001, *d* = 0.90].

Moreover, Table 3 shows the existence of a significant temporal effect: participants tend to switch less over time, and there is also a significant interaction between outcome uncertainty, the attacker's strategy, and time. Participants switch more over time when they play against Adaptive, whereas they switch less often over time when they play against Random or Minimax opponent's strategy. Furthermore, the switching rate over time depends on outcome uncertainty: people learn to switch more in Adaptive-Certain condition than in the Adaptive-Risky and Adaptive-Uncertain conditions, where the switching rate remains constant over time. Similarly, when playing against Random, the decreased switching rate is also different depending on outcome uncertainty: people learn to alternate slightly less in both the Random-Risky and Random-Uncertain conditions than in the Random-Certain condition. No difference is found across conditions when playing against Minimax strategy.

### Subjective randomization

**Figure 8:**
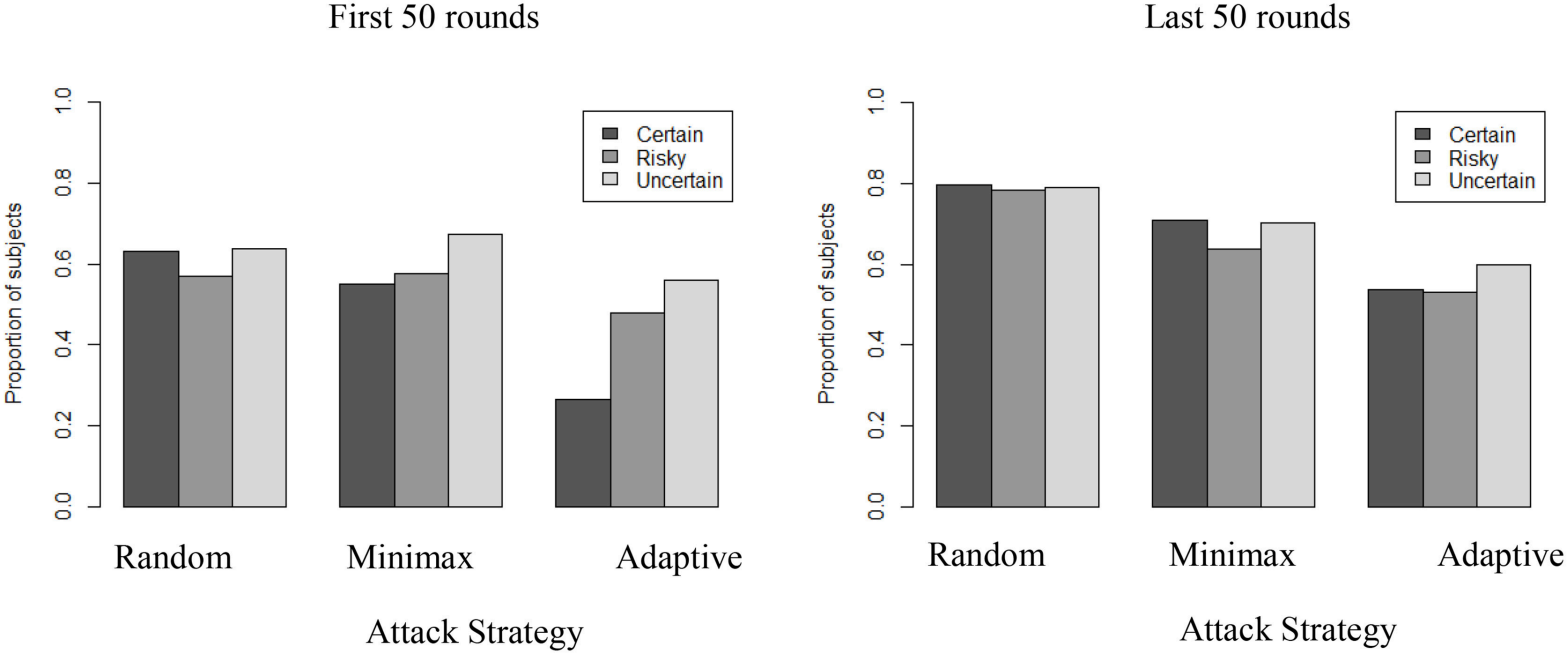
Proportion of players not exhibiting i.i.d behavior across conditions in first and last 50 rounds (Wald-Wolfowitz runs tests, *p* < 0.05).

A possible explanation for the increased rate of exploration over time against an Adaptive strategy and with full information may be a reflection of players' attempt to become unpredictable. Such explanation suggests that people may deliberately try to behave more randomly themselves when they play against an Adaptive strategy than when they play against other types of attackers. Intentional random behavior is difficult for humans to detect and perceive (e.g., Rapoport and Budescu, 1997), and many current defense strategies rely on randomization of defense resources based on game-theoretic results (Nguyen et al., 2016). To test for this explanation we used a common non-parametric test for randomness to measure independent and identically distributed (i.i.d) behavior: the Wald-Wolfowitz runs test. This test relies on the number of runs found in a time series. It compares the observed number of runs of a dichotomous variable (e.g., participant's choice between S and N in the above asymmetric security game) against the expected number of runs. More (less) runs than expected indicate the existence of over (under) alternation in the time series. Figure 8 presents the proportion of participants for whom the subjective randomization is not supported in the first (left panel) and the last (right panel) 50 rounds.

In general, a majority of participants (>50%) do not exhibit subjective random behavior and the level of uncertainty has a clear influence when playing against an Adaptive strategy, particularly in the initial rounds. Higher subjective randomness is observed in Adaptive-Certain than in either Adaptive-Risky or Adaptive-Uncertain in the first 50 rounds (*p* < 0.001), but not in the last 50 rounds. Also, in the Certain condition, subjective randomization is more common in the first 50 rounds if playing against Adaptive (26% of participants do not exhibit i.i.d.) than when playing against Minimax (55% of participants do not exhibit i.i.d.) or Random opponent strategy (63% of participants do not exhibit i.i.d.). Together with the results in the previous section, these results suggest that when playing against an adaptive strategy, more certainty leads people to behave intentionally more randomly at first (first 50 rounds), before they uncover the benefits of regular alternations (last 50 rounds).

## Discussion

Perhaps the most surprising finding is that defenders behave more optimally and with less effort (switching) when confronting attackers represented by random strategies. Defenders seem to focus their attention on how to maximize their payoffs in the long run, even if it implies losses once in a while. They seem to gradually learn that the attacker's strategy is unchanging over time. These observations are interesting as they suggest that unpredictable or random strategies often used as defense mechanisms such as “moving target defense” (Evans et al., 2011; Zhuang et al., 2013) may not be as effective against human attackers. We also find that stochastic conservative strategies such as the Minimax attack strategy are easy to exploit by human defenders, and this is relevant because such attack strategies are commonly used to generate defense schedules in real life scenarios in Stackelberg security games (Pita et al., 2010, 2012; Yang et al., 2011). Our results suggest however, that humans are able to learn to exploit this opponent's strategy, and that an adaptive strategy, which accounts for the opponent's dynamics of behavior, would be more efficient.

Another surprising finding suggests that a reduction of outcome uncertainty did not necessarily lead defenders to execute more optimal actions. Unlike Bereby-Meyer and Roth (2006), we found no significant effect of uncertainty on the speed of learning. Furthermore, this observation distinguishes asymmetric security games from existing games against nature (individual non-strategic decision problems) that reveal significant behavioral differences depending on whether information is descriptive or experiential (Hau et al., 2008; Hertwig and Erev, 2009). Defenders learn to reach good performance when playing against a random attacker where the level of outcome uncertainty seems to have only little effect. In other words, people are no more or less efficient against a random attacker when there is no uncertainty. In contrast, under uncertainty, defenders have difficulty behaving more accurately against an adaptive attacker, and they exert more effort in their attempts. Because playing optimally against a random strategy (selecting the same fixed action at every trial) can be, in principle, more accessible than playing optimally against an adaptive strategy (regularly switching actions in a precise way), one would reasonably expect the former to be more profitable. Instead, we observe that playing against these two different attack strategies can be similarly profitable under certainty. A possible interpretation is that people are naturally more reluctant to play a very basic strategy, which can be easily exploited (e.g., always playing the same action could be easily learned by the opponent), rather than a more complex one, which appears to be more robust to protect them from any type of exploitation (e.g., dynamic behavior makes it more difficult for the opponent to anticipate one's future behavior).

Finally, we find that when defenders interact with adaptive strategies and are given full information, they exert more effort to behave more intentionally in a “random manner,” as a possible attempt to be unpredictable to the attacker. This effect is particularly relevant in early interactions. Initial intentional random behavior and the following transition toward more predictable behavior may also be seen as a smooth learning exploratory strategy that helps people detect some behavioral pattern approaching actual optimal play. This may be related to a known transition from exploration to exploitation in decisions under risk (Abbasi et al., 2016).

In conclusion, our study provides helpful insights regarding the effects of uncertainty and attack behavior on a human defender behavior. Currently, most defense mechanisms inspired on game-theory assume complete information, and make unreasonable assumptions regarding rationality and cognitive plausibility of the defense mechanisms (Abbasi et al., 2015a,b). Our results suggest that humans' main defense vulnerability lies in their performance against adaptive attackers in conditions of uncertainty. Surprisingly, humans are able to handle random attacking behavior better than they are at handling adaptive attackers, suggesting that common randomized security algorithms might be less effective than adaptive human-inspired strategies for defense.

On a more practical ground, this study suggests the need to provide human defenders with more unambiguous information about possible outcomes. However, it is clear that such precise information can hardly be obtained in many real life security scenarios that strongly rely on uncertainty. To illustrate this, consider again the above situation involving a security analyst in charge of protecting a firm's network infrastructure that may be hacked by some unknown individual. In this case, the analyst has no way to know beforehand how likely a potential attacker is to counteract a defense action. The analyst simply ignores the payoffs that can result from tracking an individual that hacks the network: the hacker may indeed be more or less prepared to protect himself/herself from being identified. In order to improve the analyst's behavior in this case, our study suggests the help of a decision support system, which would provide an estimated deterministic value of the various payoffs for each possible outcome (such expected payoffs could simply be determined based on statistical data). This way, the human analyst would perceive the situation as if its description were fully known and would be more efficient at defending the firm's network. This study therefore suggests the need for increasing efforts in designing more and more efficient decision support systems that take into account the complexity of human behavior in such complex situations.

This work is only a first step toward a more general cybersecurity science. An obvious direction for future research relates to investigating behavior of trained analysts and hackers (e.g., experts) in more concrete security scenarios. Such analyses would undoubtedly help us uncover more sophisticated ways to help people protect themselves in the highly uncertain cyber world.

## Author contributions

FM contributed to the design of the study, the implementation of experimental protocols, data collection, data analyses, and write up of the manuscript. CG developed the idea of the study, contributed to the design and implementation of the study, and contributed to the writing of the manuscript.

### Conflict of interest statement

The authors declare that the research was conducted in the absence of any commercial or financial relationships that could be construed as a potential conflict of interest.
