# Supplementary material for: Security under Uncertainty: Adaptive Attackers Are More Challenging to Human Defenders than Random Attackers
Source: Front Psychol. 2017 Jun 22;8:982. doi: 10.3389/fpsyg.2017.00982 (PMC5479901; doi:10.3389/fpsyg.2017.00982)
Supplement: Supplementary file 1 [file DataSheet1.docx]

# Appendix

In the generic security game from Figure 1 if both individuals do nothing (N, N) then nobody wins or loses anything. However, if an attacker decides to hack the system while the defender does nothing (N,H), it allows him to successfully steal information of value x (x > 0). On the other hand, if a defender chooses to surveil the network from attackers who are not hacking the system (S,N), then the defender’s action (unjustified violation of the individual's privacy rights) incurs high costs y (e.g., leading to loss of clients or customers, decrease of market value, etc.). However, it is reasonable to assume that this loss does not exceed the firm’s cost of being successfully hacked (i.e., 0 < y < x). The last possible scenario consists of a defender surveiling while an attacker actively hacks the system (S,H). In this case, which characterizes a direct conflict between the two actors, the outcome is more uncertain as it depends on each player’s ability to reach their respective goal before the other. We consider that there exists some probability p (0 < p < 1) that the attack is successful (the attacker could hack into the system before the defender being able to detect a threat). This means that there is a probability 1 – p that the attack has failed (the defender was able to detect a threat before the attacker could successfully hack into the system). Note that the latter outcome provides a generous benefit z, as a result of publicly revealing its ability to successfully defend and identify the attacker (z > 0).

The uncertainty involved in (S, H) leads to defining an expected payoff for the defender that depends on the probability value p. It is clear from Figure 1 that, for any value of p, none of the defender’s choices can be weakly dominating (i.e., (S,H) is better than (N,H), and (N,N) is always better than (S,N)) . However, if considering mixed strategies (i.e., selecting S with some probability q and N with some probability 1-q), the defender can choose to behave according to the Minimax strategy that guarantees him the same minimal expected payoff, independently of the other player’s behavior. Obviously, this solution does not necessarily guarantee the highest possible payoff. In fact, although Figure 1 only includes the defender’s payoffs, we can reasonably assume that the game we are interested in is zero-sum, i.e., the potential attacker’s payoff can be simply characterized as being the converse of the defender’s (for every outcome). However, in that case, the value of probability p plays an important role in determining the defender’s best move. In fact, if p is high enough, i.e., $p\geq\frac{y+z}{x+z}$, then a potential attacker will always be better off selecting H, no matter what the defender does (N is then weakly dominated by H). Thus, assuming a zero-sum game, the defender would always be better off selecting S, which corresponds to the unique Nash equilibrium of the game. On the other hand, if 0$<p<\frac{y+z}{x+z}$, then the unique Nash equilibrium of the corresponding game consists of both players selecting their respective optimal strategies.

This game theoretic analysis has several characteristics that are highly relevant to the context of cybersecurity. First, there exists no dominant solution to be selected by any player that can offer a winning outcome regardless of what the other does. Second, the dynamic setting of the interaction suggests that the hacking action is often preceded by an *observation* phase during which the attacker aims at avoiding suspicious behavior in order to prevent the defender from anticipating the actual attack. In this case, the defender’s task becomes particularly difficult because it requires predicting future malicious behavior from past meaningless activities. Moreover, because reliable online identification (who is the real person with whom one interacts?) is difficult to achieve in a cyberworld, an attacker can hardly be permanently neutralized, and may therefore reiterate an attack multiple times without risking being caught. In this context, the defender’s main role is to protect a set of targets by counteracting various attacks. Third, given asymmetry of payoffs, the defender is often at a disadvantage compared to an attacker. In a dynamic setting (repeated play), both players following the above optimal principle in every round is the unique equilibrium (deviating from this strategy for even a single round can only bring a lower final payoff as it potentially allows the attacker to exploit this deviation). Of course, this equilibrium solution should only be seen as a reference point. Introducing a human player as a defender in this game makes the theoretical analysis more complex to track.

## Theoretical behavior

In the generic security game from Figure 1, the Minimax solution for each player can be determined as follows:

- The defender selects S with probability q$=\frac{x}{x+y+z-p(x+z)}$ (and therefore N with probability 1 - q), and he can ensure an expected payoff of ${EP}_{D}= \frac{-xy}{x+y+z-p(x+z)}$, no matter what the potential attacker does (note that, for all possible values of payoffs x, y, and z, ${EP}_{D}$< 0).
- The attacker selects H with probability r$=\frac{y}{x+y+z-p(x+z)}$ (and therefore N with probability 1 - r), and he can ensure an expected payoff of ${EP}_{A}= \frac{\mathrm{xy}}{x+y+z-p(x+z)}$, no matter what the defender does (note that, for all possible values of payoffs x, y, and z, ${EP}_{A}$ > 0).

Any strategy for the defender is considered as a best response against Minimax. Over 100 rounds, the resulting expected payoff for the defender is 66.7pts (initial 200pts – 100 × 1.33pts), no matter the response. When playing against Random, choosing S with probability 1 at every round will maximize the defender’s final payoff. In this case, the corresponding expected payoff for the defender is 250pts (initial 200pts + 100 × 0.5pt). Playing optimally against Adaptive is more complex: the defender's optimal behavior at any round r corresponds to selecting S if and only if r = 1 (Adaptive behaves as Random strategy in the first round) or $\frac{{NbT}^{r-1}}{r-1}<\frac{2}{3}$ (where r >1 and ${NbT}^{r-1}$ defines the number of times participants selected S in the r-1 previous rounds). At any round r > 1, the attacker selects H if and only if $\frac{{NbT}^{r-1}}{r-1}<\frac{2}{3}$ (and therefore N if $\frac{{NbT}^{r-1}}{r-1}\geq\frac{2}{3}$), where ${NbT}^{r-1}$ defines the number of times the human defender selected S in the r-1 previous rounds. Such an incremental strategy leads to some behavioral regularity, which consists in cyclically playing the series NTT (i.e., choosing N at some round r, and S at subsequent rounds r+1 and r+2). The corresponding expected final payoff for the defender after 100 rounds is 395.5pts.
